# High‐precision determination of lithium and magnesium isotopes utilising single column separation and multi‐collector inductively coupled plasma mass spectrometry

**DOI:** 10.1002/rcm.8020

**Published:** 2017-12-18

**Authors:** Madeleine S. Bohlin, Sambuddha Misra, Nicholas Lloyd, Henry Elderfield, Mike J. Bickle

**Affiliations:** ^1^ Department of Earth Sciences University of Cambridge Downing Street Cambridge CB2 3EQ UK; ^2^ Centre for Earth Sciences Indian Institute of Science Bangalore 560012 India; ^3^ Thermo Fisher Scientific Hanna‐Kunath‐Str. 11 28199 Bremen Germany

## Abstract

**Rationale:**

Li and Mg isotopes are increasingly used as a combined tool within the geosciences. However, established methods require separate sample purification protocols utilising several column separation procedures. This study presents a single‐step cation‐exchange method for quantitative separation of trace levels of Li and Mg from multiple sample matrices.

**Methods:**

The column method utilises the macro‐porous AGMP‐50 resin and a high‐aspect ratio column, allowing quantitative separation of Li and Mg from natural waters, sediments, rocks and carbonate matrices following the same elution protocol. High‐precision isotope determination was conducted by multi‐collector inductively coupled plasma mass spectrometry (MC‐ICPMS) on the Thermo Scientific™ NEPTUNE Plus™ fitted with 10^13^ Ω amplifiers which allow accurate and precise measurements at ion beams ≤0.51 V.

**Results:**

Sub‐nanogram Li samples (0.3–0.5 ng) were regularly separated (yielding Mg masses of 1–70 μg) using the presented column method. The total sample consumption during isotopic analysis is <0.5 ng Li and <115 ng Mg with long‐term external 2σ precisions of ±0.39‰ for δ^7^Li and ±0.07‰ for δ^26^Mg. The results for geological reference standards and seawater analysed by our method are in excellent agreement with published values despite the order of magnitude lower sample consumption.

**Conclusions:**

The possibility of eluting small sample masses and the low analytical sample consumption make this method ideal for samples of limited mass or low Li concentration, such as foraminifera, mineral separates or dilute river waters.

## 1 INTRODUCTION

Lithium and magnesium stable isotope geochemistry has the potential to provide insights into low‐ and high‐temperature geological processes such as weathering of the continental and oceanic crust (e.g.^1^, ^2^, ^3^, ^4^, ^5^, ^6^, ^7^, ^8^, ^9^, ^10^), cycling of material through the crust and mantle (e.g.^11^, ^12^, ^13^, ^14^, ^15^) and cosmochemical processes (e.g.^16^, ^17^, ^18^). The large relative mass difference between the stable isotopes of both Li and Mg (~16% difference in mass between ^7^Li and ^6^Li, and ~8% between ^26^Mg and ^24^Mg) leads to significant isotope fractionation during physical and chemical reactions,^19^ making both elements sensitive tracers for geochemical processes (e.g.^20^, ^21^). However, significant mass‐dependent isotope fractionations may occur during chemical purification and mass spectrometric measurements. It is therefore essential to avoid isotopic fractionation during chemical purification and to make appriate corrections for fractionation during analysis.

Isotope ratio determination of Li and Mg is achieved by multi‐collector inductively coupled plasma mass spectrometry (MC‐ICPMS). Due to potential isobaric interferences from doubly charged species generated in the plasma, and the effect of other matrix elements on the instrumental mass bias, it is necessary to analyse purified mono‐elemental solutions (e.g.^22^, ^23^, ^24^). This requires a multi‐step sample preparation, including the separation of Li and Mg from sample matrix through cation‐exchange chromatography. Lithium has previously been separated by using between one and four separate column procedures (e.g.^23^, ^25^, ^26^, ^27^) whereas Mg is eluted in two or three columns (e.g.^4^, ^18^, ^28^). The objective of analysing both Li and Mg on the same sample would thus require between three and seven separate column procedures. This approach is time‐consuming and increases sample blanks and the risk of incomplete sample recovery with associated isotopic fractionation.

In this paper we present a single column, one‐step elution method to separate small masses of Li and Mg from multiple sample matrices. Seawater, river water, sediment, foraminifera and rock standards with established isotopic compositions have been processed, with Li and Mg column loads varying between 0.3 and 20 ng for Li and between 1 and 70 μg for Mg to demonstrate the robustness of the method. Our technique utilises the Thermo Scientific™ NEPTUNE *Plus*™ MC‐ICPMS instrument (Thermo Scientific, Bremen, Germany) with 10^13^ Ω amplifiers, allowing sub‐nanogram samples of Li to be measured with high external precision (±0.39‰, 2σ), and consuming less than 0.5 ng per duplicate analysis. Mg isotopes are measured with 10^11^ Ω amplifiers consuming less than 115 ng Mg per duplicate analysis with a long‐term external precision of ±0.07‰ (2σ).

## 2 EXPERIMENTAL

### 2.1 Sample and standard preparation

All acids used in this study (reagent grade, Fisher Scientific, Loughborough, UK) were doubly distilled in a Teflon sub‐boiling still and prepared to the required molarity using 18.2 MΩ Milli‐Q water (Millipore, Watford, UK). The molarity of the hydrochloric acids used for column separation (0.70 N, 1.50 N and 10 N) was confirmed by titration using 1.0 M certified grade NaOH (Fisher Scientific).

**Figure 1:**
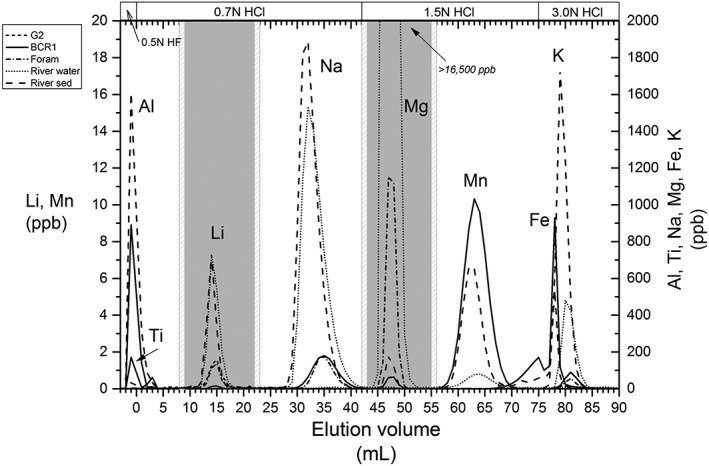
Elution curves for various sample matrices: G2 granite (short‐dashed line – only Li), BCR‐1 basalt (solid line), foraminifera calcite standard (dashed dotted line), river water (dotted line) and river sediment (dashed line). For samples with high Al and Ti load, the initial 3 mL are eluted in 0.5 N HF, and the elution volume (x‐axis) denotes the volume of HCl added. The grey boxes mark the cuts which are collected for Li and Mg isotope analysis, with the 1‐mL pre‐ and post‐cuts in grey stripes. The calibration was carried out volumetrically by collection of each millilitre of the elution. Ca, Sr and Ba elute after 100 mL. (For sample composition, see Table 1)

Samples used in this study were prepared as follows. Seawater and river water samples were evaporated to dryness at 80°C and then refluxed with 1–2 mL of concentrated aqua regia at 100°C overnight to oxidise organic matter. The samples were then evaporated to dryness and taken up in 0.7 N HCl to be loaded onto the ion‐exchange column. Sediment and rock powders were baked at 950°C for 8 h in ceramic crucibles to destroy organic matter, and then dissolved in a mixture of concentrated HNO_3_, HCl and HF (1:1:1) in Savillex© (Eden Prairie, MN, USA) screw‐top beakers on a hotplate at 110°C. Post dissolution (typically a few hours), the samples were dried down and taken up in 6 N HCl. If fluoride residues were present the sample was refluxed with concentrated HNO_3_ until a clear solution was obtained. An aliquot, generally containing 1–10 ng Li, was then diluted to 200 μL with 0.7 N HCl and loaded onto the columns. The synthetic foraminifera standard was made from pure concentrated stock solutions (ROMIL‐SpS™ super purity standards, Waterbeach, UK). The column loads of different elements for each sample matrix that were utilised to generate the column elution/calibration curves (Figure 1) are presented in Table 1.

**Table 1:** Loaded masses on column for calibration seen in Figure 1

Element	unit	Sample/matrix				
		Himalayan river water	Himalayan river sediment	BCR‐1 (basalt)	G2 (granite)	Foraminifera standard
**Al**	μg	44.5 (ng)	17.4	4.0	24.0	‐
**Ca**	μg	74.5	2.3	2.8	4.1	770.0
**Mg**	μg	30.2	0.8	1.2	1.3	3.5
**K**	μg	3.3	8.6	0.8	10.9	0.7
**Li**	ng	20.0	20.0	0.5	10.0	7.0
**Na**	μg	7.3	7.6	1.3	8.9	1.8
**Mn**	ng	2.9	68.4	84.4	68.2	70.0
**Ti**	ng	2.5	315.2	750.0	852.9	‐
**Fe**	μg	11.8 (ng)	2.9	5.4	5.5	0.1
**Li/Mg**	μg/μg	6.6 × 10^−4^	2.5 × 10^−2^	4.2 × 10^−4^	7.6 × 10^−3^	2.0 × 10^−3^
**Li/Tot**	μg/μg	1.7 × 10^−4^	5.0 × 10^−4^	3.1 × 10^−5^	1.8 × 10^−4^	9.0 × 10^−6^

### 2.2 Column chromatography

Sulphonated polystyrene cation‐exchange resins have a high load capacity (~1.7 mEq/mL dry resin) and micro‐porous gel‐type resins, such as AG50W‐X8 and AG50W‐X12 (BioRad™), are traditionally used for the chromatographic separation of Li and Mg (e.g.^4^, ^23^, ^24^, ^27^). However, the distribution coefficients for Li and Na, and those of Mg, Fe and Mn, for different strength acids and the AG50W resin are similar, especially with increasing acid strength (Table 2).^29^ Therefore, dilute acids and/or a combination of several columns are commonly utilised to fully separate both Li and Mg from other matrix elements (e.g.^4^, ^27^, ^28^). Alternatively, a mixture of dilute HCl or HNO_3_ and an organic solvent also increases the separation especially between Li and Na (e.g.^25^, ^26^, ^31^, ^32^). However, organic solvents and HNO_3_ may cause: (1) rapid degradation of the resin resulting in non‐quantifiable migration of element peaks; (2) early breakthrough of Na into the Li fraction;^30^, ^31^ and (3) rapid volatilisation of methanol, which has been hypothesised to cause element peak migration and cross contamination of Li between columns.^32^ Other strategies include initial removal of Fe from high Fe matrices by eluting through an anion‐exchange column, reducing the total matrix load.^15^, ^18^, ^33^ The peak separation of Mg, especially from Fe and Mn, and that of Li from Na, is significantly larger in the macro‐porous equivalent of the gel‐type resin – AGMP‐50 (BioRad™, Hercules, CA, USA)^34^, ^35^ (Table 2). Utilising a 3‐mL Savillex® Teflon™ ion‐exchange column with high aspect ratio (25 cm height and inner diameter of 4 mm), quantitative separation of both Li and Mg from multiple matrices is achieved in a single‐step elution.

**Table 2:** Distribution coefficients of selected elements with varying strength of HCl (0.5–2 N) for AGMP‐50 resin utilised in this study, and the AG50W‐X8 resin commonly used. Key differences include the larger separation between Li and Na, and that between Mg and Fe/Mn, using AGMP‐50

Distribution coefficients at varying acid strength						
	AG MP‐50^a^			AG 50 W‐X8^b^		
	0.5 N HCI	1.0 N HCI	2.0 N HCI	0.5 N HCI	1.0 N HCI	2.0 N HCI
**Sr**	1320	320	83	‐	60	17.8
**Ca**	850	214	57	151	41.3	12.2
**Fe (III)**	800	89	10	225	33.5	5.2
**Mn**	161	43.6	11.3	84	20.2	6
**Fe(II)**	113	30	6.9	66	19.8	4.1
**Ni**	108	27.4	6.8	70	21.9	7.2
**Mg**	89	24.8	7.2	74	20.1	6.2
**Ti**	37.8	10.7	3.9	39.1	11.9	3.7
**K**	69	34.9	17.1	29.1	13.9	7.2
**Na**	26	13.6	8.4	13.5	6.9	3.8
**Li**	9.8	5.1	3.3	8.1	3.8	2.5

a Values from Strelow.^34^

b Values from Strelow et al.^29^

#### 2.2.1 Elution protocol

**Figure 2:**
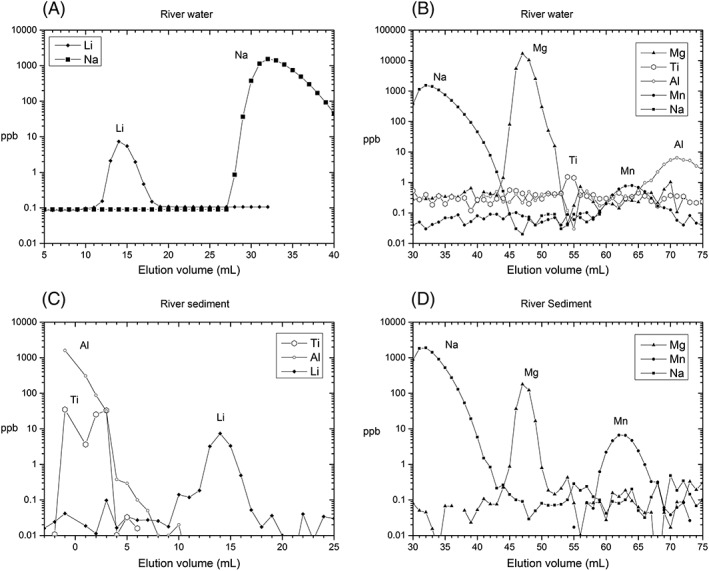
Separation of elements in the AGMP‐50 resin utilising our elution protocol, with concentration in log‐scale to magnify tailing of neighbouring elements for the river water matrix (A, B) and the river sediment matrix (C, D). Lithium is separated from Na with no peak‐tail overlap. There is a minor (although insignificant given the high Mg concentrations) tailing of Na into the Mg peak. Samples that are not eluted with initial HF (e.g. water samples and foraminifera carbonate) have Ti and Al eluting after Mg (B), compared with prior to Li when HF is used (C). Ti concentrations are low in water samples and the tailing into Mg is negligible. Note that in (A) the average Na blanks from the pre‐Na peak are plotted. Mg is clearly separated from Mn, Fe, and K (both Fe and K elute at >75 mL)

The resin was backwashed using a handheld pump and allowed to settle under gravity between each elution. This enables the resin to fully expand and uniformly distribute with homogeneous porosity between each sample elution. The columns were then conditioned with 9 mL (three column volumes) of 0.7 N HCl before being loaded with the sample (typically 2 ng Li yielding Mg masses between 1 and 70 μg). Samples were loaded in <200 μL of 0.7 N HCl, and then eluted with 9 mL of 0.7 N HCl, with the first 1 mL added incrementally in steps of 200 μL to ensure that the sample is properly loaded onto the resin. Li was then eluted in 0.7 N HCl and collected as a 13‐mL cut. A 1‐mL pre‐ and a post‐Li cut were collected to ensure that there was no Na breakthrough and that the Li peak was contained within the 13‐mL cut. Following collection of Li, the column was eluted with 18 mL of 0.7 N and an additional 1 mL of 1.50 N HCl. The Mg fraction was then collected in 12 mL of 1.50 N HCl, with a pre‐ and post‐Mg cut of 1 mL collected before and after the Mg peak. Prior to reuse, the columns were washed with 15 mL of 10 N HCl and 15 mL of 18.2 MΩ Milli‐Q water, and, depending on the loaded mass and type of sample, one more wash of 10 N HCl and water may be required, as divalent cations such as Ca, Ba and Sr are strongly retained by the AGMP‐50 resin. The same elution protocol was used for all sample matrices, with an additional 3 mL of 0.5 N HF added immediately after sample loading for rock and sediment samples with significant (weight percentage) Al and Ti.^36^ These elements may otherwise co‐elute with Mg (Figure 2) but are eluted within the first few mL of dilute HF. The full elution protocol is presented in Table 3. Following column elution the Li and Mg cuts were dried down on a hotplate at 90°C before being refluxed for 24 h with concentrated double‐distilled HNO_3_. This converts the sample into a nitrate salt and oxidises any organic matter derived from possible resin degradation. The refluxed samples were dried down on the hotplate and were then taken up in 2% HNO_3_ for analysis by MC‐ICPMS.

**Table 3:** Elution protocol for single‐step separation of Li and Mg from sample matrix

Elution cut		Volume	Reagent
	Backwash		MQ
	Condition	~ 9 mL	0.7 N HCl
E0	Load sample	200 μL	0.7 N HCl
	*(for sediments/rocks)*	*3 mL*	*0.5 N HF)*
		8 mL	0.7 N HCl
E1	Pre‐Li	1 mL	0.7 N HCl
E2	Li	13 mL	0.7 N HCl
E3	Post‐Li	1 mL	0.7 N HCl
E4	Na	18 mL	0.7 N HCl
		1 mL	1.5 N HCl
E5	Pre‐Mg	1 mL	1.5 N HCl
E6	Mg	12 mL	1.5 N HCl
E7	Post‐Mg	1 mL	1.5 N HCl
	Wash	15 mL	10 N HCl
		15 mL	MQ
		15 mL	10 N HCl
		15 mL	MQ

The elution protocol was calibrated for several different sample matrices (Table 1; Figure 1). During calibration every mL of acid was collected and analysed for concentration of cations by optical emission spectrometry (ICP‐OES, Agilent® 5100, Stockport, UK) and Li by ICP‐MS (Thermo Scientific™ ELEMENT XR™).

### 2.3 Mass spectrometry

High‐precision isotope ratio determination of both Li and Mg was performed by MC‐ICPMS at the University of Cambridge (Cambridge, UK) on the Thermo Scientific™ NEPTUNE *Plus*™ fitted with a Jet ion extraction pump. We adopted a concentration matched standard‐sample bracketing technique to correct for instrumental drift and mass bias. Each standard and sample were followed by a background instrumental blank measurement in 2% HNO_3_ matrix. A typical sequence consisted of the following measurements in the order of: blank‐standard‐blank‐sample‐blank‐standard, with blank correction performed by subtracting the average of the blank measured before and after each sample and standard. Peak‐centering was performed during each standard measurement. Both Li and Mg samples were measured in duplicate, with each measurement consisting of 33 cycles with 8.4 s integration time (total of 9 min 15 s sample analysis time) and an uptake time of 60 s.

#### 2.3.1 Li isotopic measurements

Li isotopic ratios were determined with respect to the NIST L‐SVEC standard^37^ and each analytical session included the measurement of secondary standards spiked with ^6^Li and ^7^Li (Li6‐N and Li7‐N, respectively^38^) to quantify external reproducibility. Measurements were performed using an APEX‐IR (ESI®, Omaha, NE, USA) sample introduction system with a heated spray chamber set at 140°C and a Peltier cooling coil at 2°C. Additional details of the instrumental setup are presented in Table 4. The key feature of the δ^7^Li determination method was the use of 10^13^ Ω amplifiers (Thermo Scientific) with ultra‐low electronic noise that allowed determination of precise ^7^Li/^6^Li ratios with ^6^Li and ^7^Li beam sizes of ≤35 mV and ≤0.51 V, respectively. The low baseline noise of the 10^13^ Ω amplifiers (±0.9 μV, 1σ, n = 900) gave a 4‐ to 5‐fold higher signal‐to‐noise ratio for ^6^Li beam intensities of 15–35 mV than when using 10^11^ Ω amplifiers (±4.2 μV, 1σ, n = 900). Prior to each analytical session a long baseline of 900 cycles was performed. A Savillex® C‐flow self‐aspirating nebuliser (100 μL/min), nickel Jet type sample cone and X type skimmer cone were used. This instrumental setup resulted in a ~0.4 V signal on ^7^Li, measured in the H4 cup, for a 0.4 ppb Li solution (preferred analyte Li concentration). With an uptake time of 60 s, the total sample consumption per duplicate analysis is less than 0.5 ng Li. Seawater analysed at 0.2 V yielded values indistinguishable from that analysed at 0.4 V having a total consumption of 0.2 ng Li. Lithium analysis by ICPMS techniques is plagued by rapid build‐up of Li blanks, possibly due to deposition and subsequent ionisation of Li from the skimmer cone.^31^, ^39^ Our strategy was to minimise the deposition of Li by pre‐coating the cones with alkali or alkaline earth elements.^39^ Prior to sample analysis the cones were conditioned by aspirating a 10 ppm Na solution for ~10 min. Using this “coating” technique, the Li background generally ranged from <0.5 to 3 mV, approximately 0.1–0.75% of the sample signal intensity. Without utilising the Na wash, the Li backgrounds could increase to ~100 mV, rendering it impossible to measure Li at the desired low concentration. In addition, nickel cones were preferred over platinum cones in the present study, as the latter suffered from higher and more rapidly increasing background levels, possibly due to less efficient Na‐coating on the platinum.

**Table 4:** Instrumental parameters for the analysis of Li and Mg isotopes on the NEPTUNE *Plus*™

	Li	Mg
**Preferred analyte concentration**	0.4 ppb (0.4 V on ^7^Li)	200 ppb (10 V on ^24^Mg)
**RF‐power**	1200 W	1200 W
**Guard electrode**	On	On
**Spray chamber**	APEX‐IR (Quartz)	Single pass Scott (Teflon™)
**Nebuliser aspiration rate**	100 μL/min	50 μL/min
**Injector**	1.8 mm (Platinum)	1.8 mm (Platinum)
**Sampler cone**	X (Nickel)	X (Nickel)
**Skimmer cone**	Jet (Nickel)	Jet (Nickel)
**Faraday cups**	L4, H4	L1, C, H1
**Amplifiers**	10^13^ Ω	10^11^ Ω
**Resolution**	Low	Medium
**Uptake time**	60 s	60 s
**Wash time**	90 s	90 s
**Blocks**	1	1
**Cycles**	33	33
**Integration time**	8.4 s	8.4 s
**Total analysis time per sample**	554.4 s	554.4 s
**Sample consumption**	<0.5 ng	<115 ng
**Matrix**	2% HNO_3_	2% HNO_3_
**Bracketing standard**	L‐SVEC	DSM‐3
**Secondary standard**	Li6‐N, Li7‐N	Cambridge‐1

For the Faraday cups, the letter L stands for Low mass cup, H for High mass cup and C for the Centre cup.

#### 2.3.2 Mg isotopic measurements

The ratio of the three isotopes of Mg (*viz*: ^24^Mg, ^25^Mg and ^26^Mg) were determined and bracketed against the DSM‐3 standard.^40^ Each analytical session contained the Cambridge‐1 Mg secondary standard to quantify the external reproducibility of our instrumental method. Mg isotope ratios were determined under wet plasma conditions as published work highlights that dry plasma methods may be more sensitive to residual matrix elements in the analyte (e.g.^15^, ^22^, ^42^, ^43^). A self‐aspirating Savillex® C‐flow 50 μL/min nebulizer, single pass Scott‐type Teflon spray chamber, and nickel Jet type sample cone and X type skimmer cone were used (Table 4). A 200 ppb Mg solution gave a ~10 V signal on ^24^Mg in medium resolution with this instrumental setup, giving a total sample consumption of less than 115 ng Mg per duplicate measurement.

## 3 RESULTS

### 3.1 Chromatographic separation of lithium and magnesium

**Figure 3:**
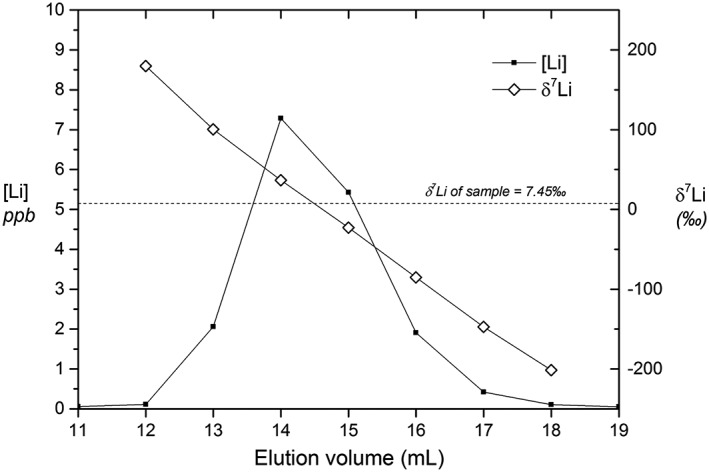
Fractionation of Li isotopes during elution of river water matrix (with δ^7^Li value of 7.45‰) through the high aspect ratio column packed with AGMP‐50 resin. The total fractionation is ±200‰. The figure illustrates the importance of quantitative recovery of the analyte during column elution

Lithium and magnesium are quantitatively separated from elements such as Na, K, Al, Ti, Mn, Fe, Ca, Sr and Ba by our column elution protocol using the AGMP‐50 resin. There is a 10 mL separation between Li and Na for Li masses ranging from 0.3 to 20 ng (Figure 1). The high Mg and Fe load for certain samples (e.g. basalts) appears to have an effect on the Li peak, as observed in previous studies.^27^ However, this occurs when the loaded Li mass is above 5 ng. Basalt samples were therefor eluted with <2 ng Li. In general, element peaks are broader for higher sample loads (Figure 1). However, the high degree of separation between different elements enables larger cuts to be collected without contamination from adjacent elements. This allowed us to follow the same protocol for all the sample matrices tested in this study. This single elution method also quantitatively separates Fe and Mn from Mg. The 1‐mL aliquots collected before and after the Li and Mg cuts were dried down and taken up in 2% HNO_3_ and measured by MC‐ICPMS on the Neptune instrument against a bracketing standard of known concentration to confirm the absence of peak tailing of Li and Mg. The pre‐ and post‐cut aliquots have concentrations of Li and Mg indistinguishable from those in the 2% HNO_3_ blank acid, confirming the quantitative recovery of the analyte within the sample aliquot. Quantitative separation is vital as mass‐dependent fractionation occurs within the column (e.g.^23^, ^25^, ^27^, ^32^, ^44^ **)**, resulting in ±200‰ fractionation of Li in our columns (Figure 3). The procedural blanks are <4 pg Li in the collected Li cut, and <1 ng Mg in the Mg cut (n = 6), ~10^−3^ and ~10^−4^ of the average loaded sample masses, respectively. The blank was measured by following the column procedure, and all subsequent post‐column steps, with a blank sample. The entire cut (13 mL for Li and 12 mL for Mg) was dried down and taken up in 1 mL 2% HNO_3_, and measured on the Neptune in concentrated form. The concentrated solutions yielded ^7^Li intensities between 3 and 4 mV (corresponding to <4 pg of Li in the cut), which is ~4 times higher than the instrument background during the measurement. The concentrated Mg cuts yielded ^24^Mg intensities between 40 and 45 mV corresponding to <1 ng of Mg.

#### 3.1.1 Peak tailing of elements with similar distribution coefficients into the Li and Mg peaks

For rock and sediment samples, the addition of dilute HF (3 mL 0.5 N HF) to the elution protocol elutes Al and Ti in the first few millilitres (Figure 2C), effectively removing a large fraction of the total matrix load. Samples not eluted with HF, such as river water and foraminifera, have Ti and Al eluting after Mg, with Ti possibly overlapping with Mg (Figure 2B). However, the concentration of Ti in river water is negligible and there are thus no detectable amounts of Ti, especially after further dilution for Mg isotope analysis. There is a slight asymmetry in the Na‐peak visible on a log‐scale (Figures 2A and 2B). The Na tailing does not drastically change between sample matrices, with similar magnitudes observed for river water samples and seawater with Na/Mg ratios ~0.25 and ~8, respectively. The Na tailing leads to co‐elution of a few ng of Na in a ~30 μg Mg peak, which is insignificant as it is further diluted by a factor of <150 before isotopic analysis on the Neptune mass spectrometer. The tailing can, however, be pronounced if the resin is not properly cleaned between successive sample passes.

### 3.2 Isotope ratio determination by MC‐ICPMS: Analyses and reproducibility of standards

The analytical fidelity of the isotope ratios of Li and Mg is dependent on their effective chromatographic separation, on appropriate reduction of the MS data, interferences and matching of sample and standard intensities and on reduction of concentrations of elements which affect mass bias. The presentation of results below evaluates the analytical protocols in terms of the potential impact on the measured isotopic ratios and confirms their effectiveness by analysis of standard reference materials.

**Figure 4:**
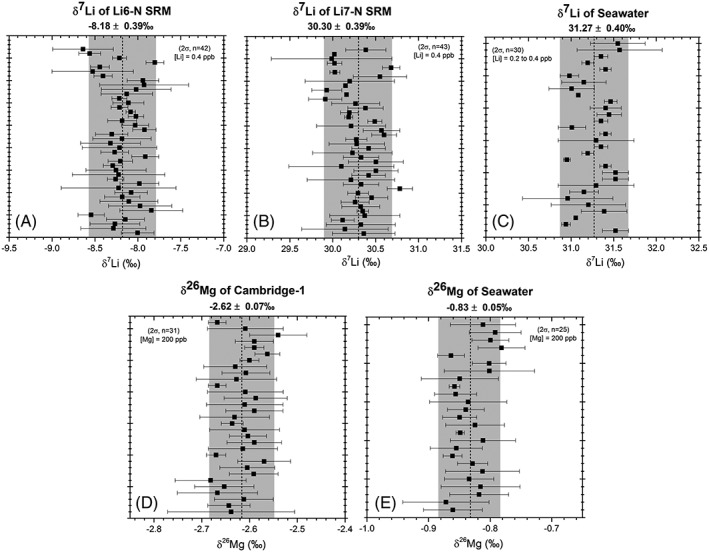
Long‐term reproducibility of measured δ^7^Li values of (A) Li6‐N (−8.18 ± 0.39‰, *n* = 42) and (B) Li7‐N (30.30 ± 0.39‰, *n* = 43) and δ^26^Mg values of (D) Cambridge‐1 (−2.62 ± 0.07‰, *n* = 31) measured during the course of 18 months. Seawater has been eluted through the columns and analysed for (C) δ^7^Li values (31.27 ± 0.40‰, *n* = 30) and (E) δ^26^Mg values (−0.83 ± 0.05‰, *n* = 25). The grey bands show the 2 standard deviations of the long‐term mean of the sample population

The isotopic ratios of Li and Mg are expressed in the δ‐notation (‰) by the convention:

δHX=XH/XLsampleXH/XLstandard−1×1000

where X is either Li or Mg, H is the heavy isotope and L the light isotope. Lithium samples are normalised to NIST SRM 8545 L‐SVEC and Mg to DSM‐3. Long‐term average δ^7^Li values of Li6‐N and Li7‐N secondary standards are −8.18 ± 0.39‰ (2σ, *n* = 42) and 30.30 ± 0.39‰ (2σ, *n* = 43), respectively (Figure 4). These are within the range of reported values of −8.9 to −8‰ and 30.2 to 30.4‰ for Li6‐N and Li7‐N, respectively.^6^, ^7^, ^38^ The Cambridge‐1 Mg standard yields a long‐term average δ^26^Mg value of −2.62 ± 0.07‰ (2σ, *n* = 31), identical to published values (e.g.^4^, ^34^, ^41^, ^45^ **)**. Seawater processed through the columns gives mean δ^7^Li values of 31.27 ± 0.40‰ (2σ, *n* = 30 times through columns) and δ^26^Mg values of −0.83 ± 0.05‰ (2σ, *n* = 25 times through columns), indistinguishable from accepted values of 31.0 ± 0.5‰ (e.g.^45^) and −0.83 ± 0.09‰.^46^, ^47^

#### 3.2.1 δ^7^Li and δ^26^Mg values of standard reference materials

The isotopic ratios of Li and Mg are widely used within the geosciences, with application to both low‐ and high‐temperature geochemical processes. Column chromatography methods are usually adapted to the preferred type of sample matrix with rock and sediment samples, with a high cationic content, requiring careful handling during column elution. To validate the column protocol and analytical technique described in this study we analysed a set of geological reference standards with published Li and Mg isotopic compositions. The results are in excellent agreement with published and accepted values (Tables 5 and 6).

**Table 5:** δ^7^Li values of multiple reference standards

	δ^7^Li	2σ	*n*	Loaded mass	Reference
	(‰)	(‰)		(ng)	
**BIR‐1**	**3.49**	**0.01**	**1**	**2**	**This study**
*Basalt*	3.30	0.60	5		54
	3.39	0.77	9		55
	3.9	1	4		56
**BCR‐2**	**2.82**	**0.13**	**2**	**1‐2**	**This study**
*Basalt*	3.50	0.20	22		54
	3.10	0.90	9		57
	2.6	0.3	18	10	58
	2.6	0.3	19	10	59
**BHVO‐2**	**4.76**	**0.29**	**6**	**1‐2**	**This study**
*Basalt*	4.40	0.80	11		57
	4.9	1.04	11	374	60
	4.7	0.2	31	10	59
	4.2	0.5	17		61
	4.7	0.2	31	10	15
	4.5	0.27	13		62
	4.8	0.2	15		63
**SGR‐1b**	**4.96**	**0.62**	**6**	**2‐5**	**This study**
*Shale*	3.6	0.4	3	20	64
	4.73	0.7	3	200‐400	65
**JCp‐1**	**20.27**	**0.41**	**4**	**0.3**	**This study**
*Aragonite*	20.16	0.2	5	1.2	24
**Seawater**	**31.27**	**0.4**	**30**	**0.3‐5**	**This study**
	30.55	0.45	15	**‐**	66
	30.88	0.12	46	1.2	24
	31.01	0.54	90	1	9
	31.1	0.2	31	2	31
	31.2	1.8	28	3‐15	67
	31.8	1.9	15	40	26

*n* is the number of analyses, which equals the number of column separations for this study. Studies using MC‐ICPMS are preferentially referenced for comparison. In addition, for commonly used standards, studies with 10 or more analyses are included (for a more comprehensive list of references, see http://georem.mpch‐mainz.gwdg.de).

**Table 6:** δ^26^Mg and δ^25^Mg values of multiple reference standards

	δ^26^Mg	2σ	δ^25^Mg	2σ	*n*	Reference
	(‰)	(‰)	(‰)	(‰)		
**BIR‐1**	**−0.31**	**0.04**	**−0.17**	**0.05**	**2**	**This study**
*Basalt*	−0.22	0.06	−0.10	0.02	11	68
	−0.27	0.33	−0.18	0.18	14	69
	−0.29	0.01	−0.15	0.01	16	70
**BCR‐2**	**−0.26**	**0.02**	**−0.13**	**0.05**	**4**	**This study**
*Basalt*	−0.16	0.01	−0.08	0.02	35	68
	−0.26	0.08	−0.13	0.05	54	71
	−0.32	0.15	−0.16	0.07	12	72
	−0.30	0.19	−0.16	0.11	31	69
	−0.26	0.13	−0.13	0.07	134	73
	−0.30	0.11	−0.15	0.07	18	41
	−0.30	0.08	−0.16	0.09	28	74
	−0.19	0.07	−0.09	0.07	15	70
**BHVO‐2**	**−0.26**	**0.07**	**−0.14**	**0.04**	**6**	**This study**
*Basalt*	−0.22	0.04	−0.10	0.03	14	68
	−0.20	0.07	−0.10	0.05	54	71
	−0.31	0.19	−0.16	0.11	30	69
	−0.19	0.07	−0.10	0.03	10	75
**AGV‐2**	**−0.16**	**0.08**	**−0.08**	**0.05**	**3**	**This study**
*Andesite*	−0.12	0.03	−0.06	0.03	19	68
	−0.24	0.24	−0.14	0.13	28	69
	−0.22	0.18	−0.12	0.08	15	73
**G2**	**−0.08**	**0.02**	**−0.03**	**0.04**	**1**	**This study**
*Granite*	−0.15	0.07	−0.08	0.06	12	44
	−0.13	0.05	−0.07	0.04	34	68
	−0.22	0.25	−0.07	0.14	16	41
**SDC‐1**	**−0.07**	**0.02**	**−0.03**	**0.01**	**1**	**This study**
*Mica schist*	−0.11	0.03	−0.06	0.05	4	44
**Sco‐1**	**−0.85**	**0.05**	**−0.43**	**0.01**	**3**	**This study**
*Shale*	−0.91	0.04	−0.48	0.03	‐	76
	−0.89	0.08	−0.47	0.05	4	44
	−0.94	0.08	−0.50	0.06	1	77
**SGR‐1b**	**−0.97**	**0.03**	**−0.52**	**0.03**	**3**	**This study**
*Shale*	−1.00	0.08	−0.51	0.03	4	44
	−0.98	0.12	−0.50	0.06	3	78
**JCp‐1**	**−2.00**	**0.12**	**−1.05**	**0.07**	**25**	**This study**
*Aragonite*	−2.02	0.11	−1.05	0.06	15	79
	−2.01	0.22	−1.05	0.12	37	78
**Seawater**	**−0.83**	**0.05**	**−0.43**	**0.02**	**25**	**This study**
	−0.84	0.06	−0.43	0.04	102	44
	−0.83	0.11	−0.43	0.06	49	47
	−0.82	0.01	−0.43	0.01	26	46

*n* is the number of analyses, which equals the number of column separations for this study. Studies using MC‐ICPMS are preferentially referenced for comparison. In addition, for commonly used standards, studies with 10 or more analyses are included (for a more comprehensive list of references, see http://georem.mpch‐mainz.gwdg.de).

## 4 DISCUSSION

### 4.1 Plasma‐based ^12^C^14^N^+^‐interference on ^26^Mg

**Figure 5:**
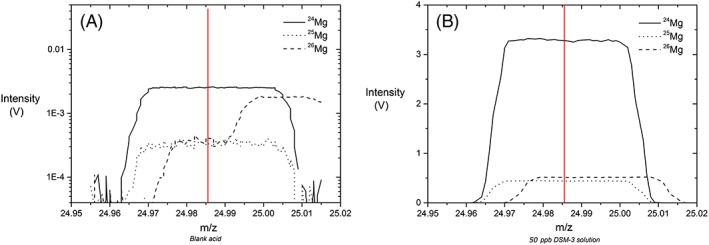
Offset H1 cup (^**26**^Mg) towards higher mass to avoid CN interference. (A) CN interference (~0.9 mV) seen in blank acid, located on the right hand shoulder of the ^**26**^Mg peak (dashed line). (B) Peak‐centering (red vertical line) is performed in DSM‐3 standard on ^**25**^Mg (dotted line) [Color figure can be viewed at wileyonlinelibrary.com]

Accurate determination of Mg isotope ratios may suffer from isobaric interference of carbon nitride, ^12^C^14^N^+^, on ^26^Mg (Teng and Yang^41^) (Figure 5A). All the Mg measurements in this study were performed in medium resolution with an offset of the H1 cup (first high mass cup, used for the determination of ^26^Mg) towards a higher mass (Figure 5). The CN interference sits on the right‐hand shoulder of the ^26^Mg‐peak and an offset of the H1 cup towards a higher mass, combined with peak‐centering on ^25^Mg, quantitatively avoids the CN interference on ^26^Mg.

### 4.2 Sample‐standard concentration matching

**Figure 6:**
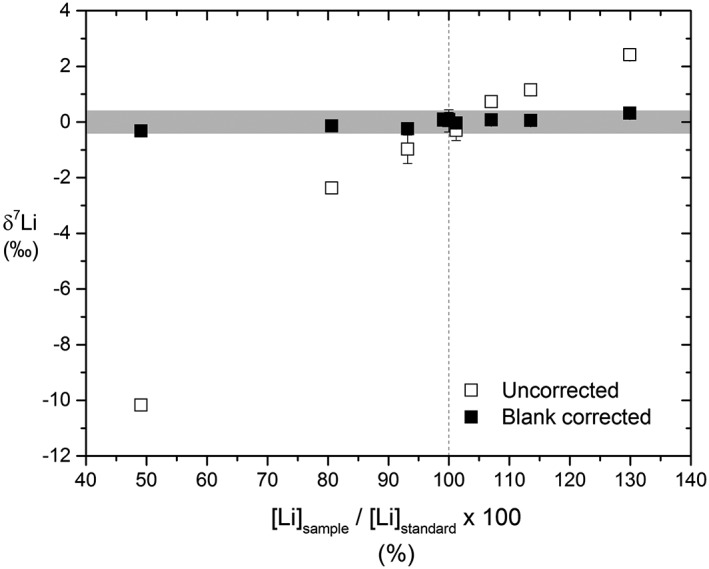
Uncorrected (open squares) and blank corrected (filled squares) δ^7^Li values of L‐SVEC with varying sample/standard concentration ratios. The grey field marks the external reproducibility in this study (±0.39‰). Blank corrected values all fall within this field

Several studies have highlighted the importance of accurate concentration matching between samples and the bracketing standard during isotope analysis. Instrumental backgrounds with very light δ^7^Li compositions (−200‰) have been shown to cause analytical artefacts on measured ^7^Li/^6^Li when the concentrations of the bracketing standard and sample have deviated by more than 50%.^24^, ^48^, ^49^, ^50^ Concentrations of samples and standards were therefore matched to within ±10% of each other in this study. Especial care was taken for Li as the ^7^Li beam intensities were close to the saturation voltage of the amplifiers (0.51 V with 10^13^ Ω resistors). To test the effects of mismatched concentrations of our instrumental method, L‐SVEC was measured at varying concentrations against the bracketing L‐SVEC standard (Figure 6). The resulting δ^7^Li values remain within the external precision of the Li method (±0.39‰) for all tested sample/standard concentration ratios (Figure 6). However, the large mass bias observed for the raw δ^7^Li values (e.g. −10‰ at sample/standard ratio of 0.5) confirms previous studies showing that instrumental backgrounds have light δ^7^Li compositions.^24^, ^48^, ^49^, ^50^ Further, it highlights the importance of accurate blank correction.

### 4.3 Matrix element effects

**Figure 7:**
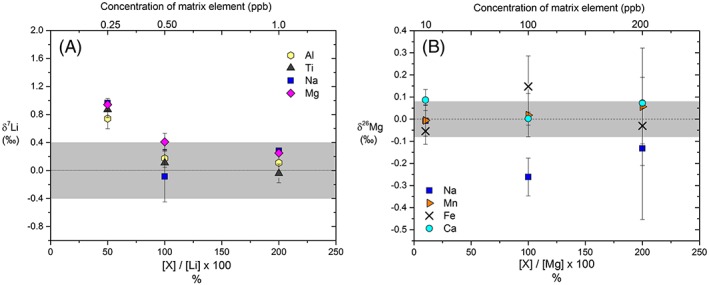
Effect of contaminant matrix elements on (A) δ^7^Li values of L‐SVEC and (B) δ^26^Mg values of DSM‐3. Li was analysed at 0.5 ppb and Mg at 100 ppb in this test. All values are blank corrected by subtracting the measured value of the blank preceding and following each sample and standard. The grey fields mark the external reproducibility in this study (±0.39‰ for δ^7^Li measurements and ±0.07‰ for δ^26^Mg measurements) [Color figure can be viewed at wileyonlinelibrary.com]

**Figure 8:**
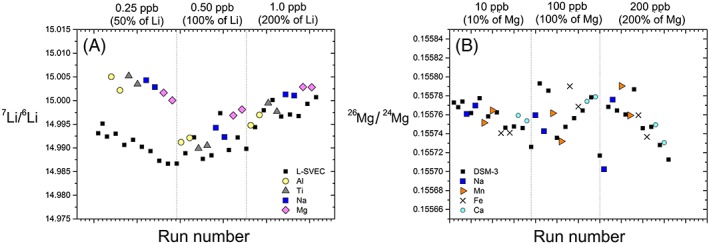
Effect of contaminant matrix elements on (A) δ^7^Li values of L‐SVEC and (B) δ^26^Mg values of DSM‐3 with run number during the analytical sequence. Li was analysed at 0.5 ppb and Mg at 100 ppb in this test. All values are blank corrected by subtracting the measured value of the blank preceding and following each sample and standard. See text for discussion [Color figure can be viewed at wileyonlinelibrary.com]

The presence of matrix elements in the analyte may degrade the accuracy of Mg isotope ratio determinations in dry plasma conditions (e.g.^22^, ^41^, ^42^), although instrumental mass bias in wet plasma appears to be less sensitive.^15^, ^51^, ^52^ Matrix‐induced mass bias is similarly recognised for Li isotope ratio determinations, especially when dry plasma is generated using an Aridus® membrane‐containing desolvator.^31^, ^49^ The presence of matrix elements with intensities twice that of the Li beam has shown detectable changes in mass fractionation characterised by a decrease in δ^7^Li values by up to 3‰.^26^, ^32^ However, the use of ESI® APEX‐IR as a desolvator has been shown to produce stable δ^7^Li values of L‐SVEC doped with Mg, Al or Na up to ten times the concentration of Li.^24^ As Li is measured at very low concentrations/voltages in this study the presence of small amounts of contaminant elements can have a disproportionate effect. Samples analysed for Li and Mg isotopes were therefore scanned for contamination from Na, Al, Ca and Fe prior to isotope analysis, and always had amounts indistinguishable from those of the bracketing standard and wash solution. However, to test the effects of possible contamination, matrix element doped solutions of L‐SVEC and DSM‐3 at contaminant/sample ratios of 0.1, 0.5, 1 and 2 were analysed against pure L‐SVEC and DSM‐3 solutions (Figures 7A and 8A). Elements that elute close to the Li and Mg peaks during column separation were prioritised for the doping test. In addition, Mg doping for Li was carried out, as Mg is a common contaminant in plastic vials.

Several 0.5 ppb L‐SVEC solutions were individually doped with Na, Mg, Al and Ti (single‐element high‐purity ICPMS standards) at concentrations of 0.25, 0.50 and 1.0 ppb, with resulting contaminant/Li ratios ranging from 0.5 to 2. The introduction of matrix elements (0.25 ppb, 50% of the measured Li concentration) caused a decrease and destabilisation in the ^7^Li/^6^Li ratio of the bracketing contaminant‐free L‐SVEC standard (Figure 8A). The shift in mass bias could be caused by a change in the surface chemistry of the Apex and subsequent re‐equilibration, or an initial increase in secondary ionisation of Li off the skimmer cone as a result of substitution of Li by matrix elements; however, this is speculative as further tests were not carried out. The lowering of the ^7^Li/^6^Li ratio of the bracketing standard coupled with biased transmission of ^7^Li in the matrix‐doped solutions (possibly due to increased space‐charge effects) led to the initial δ^7^Li value of matrix‐doped L‐SVEC being ~ +1‰ higher than the true value. However, the system re‐equilibrated after ca 3 h analysis time, with the δ^7^Li values being within the external precision of our method even at contaminant/Li ratios of 2. The destabilisation that occurred at the first introduction of contaminant elements highlights the importance of careful sample handling during chemical purification and preparation, as a small amount of matrix elements may have a long lasting effect before re‐equilibration occurs. We also observe no systematic offset in the measured δ^7^Li values driven by the choice of doping element.

**Figure 9:**
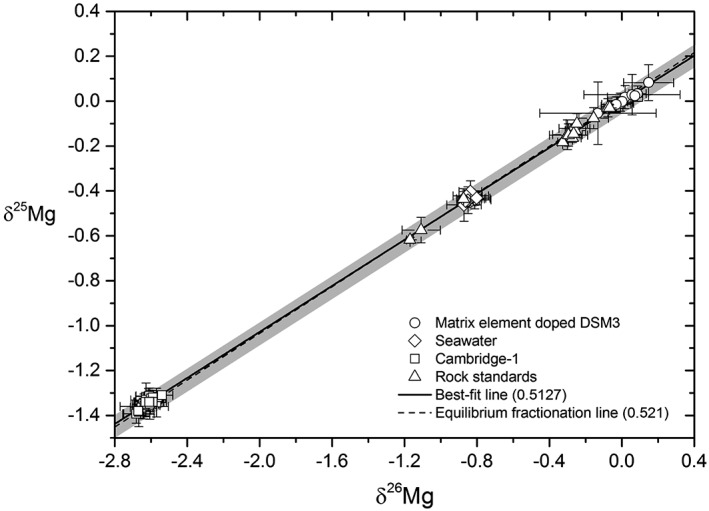
A cross plot of δ^25^Mg and δ^26^Mg values of samples analysed in this study.^53^ The solid black line is the best‐fit linear regression through the data set (slope = 0.5127, R^2^ = 0.9997) and the dashed line is the theoretical equilibrium fractionation line^53^ (slope = 0.521). DSM‐3 solutions doped with matrix elements (circles) do not show observable deviation from the regression line but do, however, suffer from larger instrumental uncertainty than other purified samples

To test the effects of matrix element contamination on Mg isotopic ratios, DSM‐3 at 100 ppb was doped with Na, Ca, Mn and Fe at 10, 100 and 200 ppb yielding contaminant/Mg ratios of 0.1, 1 and 2. We observe that the scatter in the measured Mg isotope ratio increased with the addition of contaminant elements (Figures 7B and 8B). The presence of Na lowers the δ^26^Mg value, whereas Fe appears to increase the measured δ^26^Mg value, although we did not observe any discernible trend in δ^26^Mg values with the added mass of elements. Addition of Mn and Ca has no effect on the average δ^26^Mg value at the concentrations utilised in the present experiment. However, the instrumental precision is reduced with the addition of the matrix elements, especially Ca. The values for the doped DSM‐3 solutions do not deviate significantly from the mass‐dependent fractionation line (with Δ^25^Mg' values^53^ within ±0.04, grey field in Figure 9), and Ca concentrations up to 200 ppb did not cause systematic interference on ^24^Mg when the ratios were measured in medium resolution. The results from both the Li and the Mg experiments highlight the importance of quantitative separation during column separation, and care during post‐column processing.

## 5 CONCLUSIONS

A single‐step cation‐exchange column method has been established for the combined separation of trace levels of Li and Mg from natural sample matrices. We utilise the high separation factors between Li and Na, Mg and Fe, Mn and K, in the macro‐porous resin AGMP‐50, combined with a high aspect ratio column for the quantitative separation of Li and Mg after a single elution. The cumulative blanks are low (<4 pg Li and <1 ng Mg) allowing sub‐nanogram amounts of Li to be processed. Li was typically loaded at between 0.3 and 20 ng yielding Mg masses between 1 and 70 μg. Li and Mg isotopic ratios were measured by MC‐ICPMS on the Thermo Scientific™ NEPTUNE *Plus*™. Li isotope analyses were performed utilising 10^13^ Ω amplifiers on the Faraday collectors, which allowed accurate and precise determination of isotopic ratios at ^7^Li ion beams of <0.51 V, with a total sample consumption of <0.5 ng Li per duplicate analysis. Mg was measured in medium resolution with 10^11^ Ω amplifiers at ~10 V signal on ^24^Mg with a total sample consumption of <115 ng Mg per duplicate analysis. The long‐term external precision (2σ) is ±0.39‰ and ±0.07‰ for δ^7^Li and δ^26^Mg values, respectively, determined by repeated measurements over 18 months of secondary standards (Li6‐N, Li7‐N and Cambridge‐1). The δ^7^Li and δ^26^Mg values obtained for several geological reference standards are in excellent agreement with published values. Seawater has been eluted with Li masses ranging from 0.3 to 5 ng, yielding an average δ^7^Li value of 31.27 ± 0.40‰ (*n* = 30) and δ^26^Mg value of −0.83 ± 0.05‰ (*n* = 25). The possibility of eluting small masses and the low analytical sample consumption make this method ideal for samples of limited mass or low Li concentration, such as foraminifera, mineral separates or dilute river waters.
